# Cat epididymal semen cryopreserved with and without vitamin E: effect on sperm parameters and lipid peroxidation

**DOI:** 10.21451/1984-3143-AR2018-0001

**Published:** 2018-12-05

**Authors:** Beatrice Ingrid Macente, Raquel Ribeiro Gutierrez, Maricy Apparício, Cristiano de Carvalho Balieiro, Cleber Fernando Menegasso Mansano, Marcelo Maia Pereira, Juliana Corrêa Borges-Silva, Eliandra Antonia Pires-Buttler, André Luis Batista Galvão, Gilson Hélio Toniollo, Gaia Cecília Luvoni, Maria Giorgia Morselli, Wilter Ricardo Russiano Vicente

**Affiliations:** 1 Universidade Estadual Paulista (UNESP), Jaboticabal, São Paulo, Brazil.; 2 Faculdade de Jaguariúna (FAJ), Jaguariúna, São Paulo, Brazil.; 3 Università degli Studi di Milano (UNIMI), Milano, Italy.

**Keywords:** antioxidant, feline, oxidative stress, sperm, α-tocopherol

## Abstract

The aims of this study were to investigate: 1) if the addition of α-tocopherol (vitamin E) in three concentrations (0.3, 0.6 and 0.9 mM) is able to preserve spermatozoa integrity after thawing and 2) the effect of α-tocopherol supplementation on lipid peroxidation. Fifty four domestic cats were used in this study constituting 18 pools (3 cats per pool). Each pool was submitted at four experimental groups: group 0 (control) – epididymal sperm were frozen with a commercial Botucrio^®^ extender; group 0.3, group 0.6 and group 0.9 – the extender was supplemented with 0.3, 0.6 and 0.9 mM of α-tocopherol, respectively. Each semen sample was evaluated for motility, progressive forward motility, morphology, sperm viability (plasma membrane integrity-PMI), hypo-osmotic swelling test (HOST), before and after thawing. The evaluation of lipid peroxidation reaction by Thiobarbituric Acid Reactive Substances (TBARS) test was performed on thawed semen only. Results demonstrated that there was no significant difference between control and the three α-tocopherol groups with regards to motility and progressive motility after thawing (P > 0.05). As expected, in fresh samples viability was significantly higher than in all the cryopreserved groups in which there was no positive influence of any of the α-tocopherol concentration used. Lipid peroxidation was higher in the supplemented groups 0.6 and 0.9 mM of α-tocopherol than in control and in 0.3 mM group. In conclusion, the addition of α-tocopherol to the commercial extender had no positive influence on reduction of lipid peroxidation. This topic deserves further investigations to better understand the effect of cryopreservation procedures on epididymal spermatozoa and to establish adequate strategies to counteract sperm cryodamages.

## Introduction

The domestic cat is an excellent research model for wild felids threatened by extinction and for the study of human pathologies, especially those concerning teratozoospermia ( Luvoni *et al*., 2003 ). Different protocols of cryopreservation for both ejaculated and epididymal cat spermatozoa have been tested ( Buranaamnuay, 2017 ). The advantage of using epididymal spermatozoa is that it allows the preservation of genetic material from males submitted to castration and from those that die unexpectedly ( Thuwanut *et al*., 2008 ).

Although feline semen conservation has brought encouraging results, an ideal diluent has not yet been defined ( Zambelli *et al*., 2010 ; Buranaamnuay, 2017 ), once thawed, frozen spermatozoa are usually associated with extensive acrosome loss and reduced motility ( Melo *et al*., 2007 ).

It has been described that during cryopreservation, spermatozoa are subjected to oxidative stress, an imbalanced condition between reactive oxygen species (ROS) occurrence and naive antioxidant defense mechanisms, which has been shown to compromise sperm quality ( Thuwanut *et al*., 2008 ). In order to minimize the toxic effects of ROS to spermatozoa, researchers have added antioxidants or antioxidative enzymes to sperm extenders of several species with contradictory results ( Breininger *et al*., 2005 - boar; Michael *et al*., 2007 - canine; Satorre *et al*., 2012 - boar). In cats, the addition of cysteine or trolox (a water soluble vitamin E analogue) to Tris egg yolk extender improved motility, membrane and DNA integrity, but had no effect on acrosome integrity ( Thuwanut *et al*., 2008 ). On the other hand, supplementation of Tris egg yolk extender with glutathione peroxidase was not efficient to protect spermatozoa from lipid peroxidation reaction ( Thuwanut *et al*., 2010 ). Thus, there is a need for further investigation on the effects of different antioxidants, associations and concentrations on sperm quality of feline species.

The addition of α-tocopherol, the most potent antioxidant compound of vitamin E ( Michael *et al*., 2007 ), to semen extender and the effect on lipid peroxidation have not yet been evaluated for cryopreservation of cat spermatozoa and so, the present study was performed to investigate: (a) whether α-tocopherol supplemented in the cryopreservation extender would improve the quality of cat epididymal spermatozoa after thawing, and (b) the effect of α-tocopherol supplementation on lipid peroxidation. We hypothesized that vitamin E may act efficiently as an antioxidant, reducing cellular damage and lipid peroxidation during cryopreservation.

## Materials and Methods

All chemicals in this study were purchased from Sigma-Aldrich Chemical Company (St. Louis, MO, USA) unless otherwise stated.

### Experimental design

A total of fifty four cats were included in the study. In order to have an appropriate volume for the freezing and post thawing evaluation, epididymal spermatozoa from three cats were pooled every time (n = 18 pools). Each pooled sample was evaluated before freezing and after thawing for sperm morphology, concentration, subjective motility, forward progressive motility, viability, hypo-osmotic swelling test (HOST). The evaluation of lipid peroxidation through Thiobarbituric Acid Reactive Substances assay (TBARS) was performed only after thawing. Then, samples were equally divided into four groups: (group 0) diluted into commercial Botucrio^®^ extender (BC), and diluted in BC with three concentrations of α-tocopherol (group 0.3, group 0.6 and group 0.9 mM). The commercial BC extender was initially formulated for stallion semen ( Melo *et al*., 2007 ) and it has been tested in dog´s semen with satisfying results ( Lopes *et al*., 2010 ). This extender was adopted also because in our previous studies a beneficial effect on cat spermatozoa membrane integrity was observed after thawing ( Macente *et al*., 2012 ) and the use of a commercial product has the advantage to be standardized and easy to perform, and it is practical for outside use when no laboratory condition is available. In addition, the concentrations of the supplemented α-tocopherol were based on previous studies of our laboratory (Savi *et al*., 2012, São Paulo State University - UNESP, Jaboticabal, São Paulo: unpublished data) and on experiments reported in the literature ( Michael *et al*., 2007 ; Lopes *et al*., 2010 ).

Each epididymal sperm sample was packed in straws and then cooled at 4°C within 1 h. After, the straws were cryopreserved in nitrogen vapor for 20 min and then, submerged in liquid nitrogen. The thawing was performed in water bath at 70ºC during 8 sec.

### Animals and collection of epididymal spermatozoa

Privately owned mixed breed male cats (n. 54) between 1 and 5 years of age and weighting between 2.8 and 5.4 kg were enrolled in this experiment. Animals were subjected to routine sterilization after being considered clinically healthy. After orchiectomy, ducts deferens were tied and testes were placed into physiological saline at room temperature and immediately transported to the laboratory.

Within 1 h of collection, blood vessels were removed and epididymis and deferent ducts were isolated using a Halsted Mosquito forceps. Then, spermatozoa were collected by a modified compression technique. Briefly, deferent ducts were placed over a Petri dish inclined at an angle of 45° containing 250 μl of Ringer’s solution at room temperature ( Martins *et al*., 2009 ). Then, they were compressed (squeezed) using a glass microscope slide in one direction in order to release spermatozoa into the ringer solution and the sample was transferred into a 1.5 ml tube. The Petri dish was washed with additional 250 μl of ringer solution to avoid loss of material and the content was again transferred to the tube.

### Sperm evaluation

#### Sperm concentration, motility and morphology

The samples were stabilized in a hotplate at 38°C for 10 min before the procedure. To evaluate sperm motility and progressive motility, an aliquot of the sperm sample was placed on a pre-warmed glass slide, covered with a warmed cover slip and subjectively evaluated under a phase-contrast microscope at 200x. Motility was estimated as the percentage of motile spermatozoa (0-100%). Forward progressive motility was evaluated based on the type of forward movement of sperm cells (scale bar 0-5), in which 0 is immotile spermatozoa; 1 is very slow erratic motion, no forward progressive movement; 2 is slow forward progressive movement; 3 is moderate straight line movement across the field; 4 is good forward progressive movement and 5 is rapid forward progression. Only samples exhibiting motility ≥70% and forward progressive motility ≥3 were included in this study.

Samples were then centrifuged at 1200 x g for 10 min. After removal of the supernatant, an aliquot of 5 μl was resuspended in 200 μl of distillate water to determine sperm concentration and spermatozoa were counted in 25 squares of a Neubauer hemocytometer. An ideal concentration of 5x10^6^ spermatozoa/straw of 250 μl has been stablished. Morphology was determined by using eosin yellow dye and 200 spermatozoa were evaluated under a phase-contrast microscope (x1000). Morphological abnormalities were classified according to Axnér *et al*. (2004) with some modifications in: minor head abnormalities, midpiece implantation abnormalities, minor tail abnormalities (bent tail, terminally coiled tail), distal droplets, major head abnormalities, midpiece defects, major tail abnormalities (strongly coiled or folded tails), proximal droplets, teratogenic defects, coiled tail with droplet.

#### Plasma membrane integrity (viability-PMI) and membrane functionality (HOST).

Viability was determined by the eosin stain technique, in which 5 μl of the sample was mixed with 0.1 μl of eosin yellow solution. For each sample, 200 cells were assessed using an optic microscope at 1000x magnification under mineral oil. Dead spermatozoa stained red, whereas the absence of color indicate spermatozoa membrane integrity.

The hypo-osmotic swelling technique was performed according to the test described by Jeyendran *et al*. (1984), in which sperm were incubated in a hypo-osmotic solution (150 mOSM of fructose and sodium citrate) at 37°C for 60 min. The percentage of spermatozoa with swollen sperm tails (intact sperm cell) was determined in phase contrast microscope at 40x magnification. Two hundred spermatozoa were evaluated for each sample and classified by the shape of the tail. The percentage of spermatozoa that showed typical tail abnormalities indicative of swelling was recorded; then, to calculate the real number of spermatozoa that react to the hypo-osmotic test, the proportion of spermatozoa with swollen sperm tails was subtracted by the number of altered spermatozoa with curled tail (determined during morphological evaluation). This second test to assess membrane integrity was performed because until that present study, the hypo-osmotic test was reporting in only one study ( Comercio *et al*., 2013 ).

#### Lipid peroxidation reaction

Lipid peroxidation reaction was determined by using Thiobarbituric Acid Reactive Substances test (TBARS) following the protocol described by Buege and Aust (1978) , with modifications. A 200 μl aliquot of the frozen sample from each group was used. TBARS value was determined by mixing 100 μl of the sample with 2 ml of TCA-TBA-HCL stock solution (15% v trichloroacetic acid; 0.25N hydrochloric acid, 0.375% w/v thiobarbituric acid). The remaining 100 µl were transferred to tubes containing 2 ml of BHT solution (Stock solution + 50 mM BHT and 0.24 mM heptahydrate ferrous sulfate ( Paya *et al*., 1992 ). These tubes were incubated at 100°C for 15 min and then cooled in ice for additional 15 min. After this, samples were centrifuged at 1200 x g for 15 min and the supernatant was collected and TBARS were quantified on a spectrophotometer (Thermo Fisher Scientific – Genesy 20, China), using wavelength of 532 nm.

The amount of free radicals produced by the sample is represented as FR while the maximum production of free radicals within the sample is represented as GS. The difference between GS and FR is the antioxidant power (TBARS production). The highest TBARS value corresponds to the best antioxidant power. The results of lipid peroxidation were expressed in TBARS/ 20x10^6^ spermatozoa per ml.

#### Freezing and thawing of spermatozoa

After determining sperm concentration, the number of straws and the amount of extender was established. Only pooled samples with enough volume to be distributed into eight straws (two per group) were included in this study. The pellet was resuspended in 400 µl of the commercial BC extender (without antioxidant). The diluted sample was then divided in four aliquots that corresponded to each experimental group, of which one was control (group 0) and three were with α-tocopherol at the following concentrations: 0.3, 0.6 and 0.9 mM for group 0.3, group 0.6 and group 0.9 respectively. The aliquots were package into 0.25 ml straws and were sealed (with a final concentration of 20 x10^6^ spermatozoa/ml). The straws were incubated at 4^o^C for 1 h and frozen 6 cm above the level of liquid nitrogen for 20 min before being plunged into liquid nitrogen for storage.

For the post thaw evaluation, the straws were thawed at 70°C for 8 sec ( Cocchia *et al*., 2009 ) and maintained at 38ºC. The samples were evaluated again for motility, progressive motility, morphology, PMI, HOST and TBARS.

#### Statistical analysis

Data were analyzed using Statistical Analysis System (2008). Percentages were arcsine-transformed (to normalize distributions). Differences among means were evaluated with ANOVA followed by Tukey test to identify the significant differences. Values were presented per percentage or as means ± SD and the level of significance was set at P < 0.05.

## Results

### Subjective motility and progressive forward movement

Total motility did not differ among samples after semen dilution and ranged from 70 to 90 (75 ± 6.1%). The motility in frozen-thawed samples differed from the fresh samples ( Tab. 1 ).

**Table 1:** Mean (± standard deviation) sperm motility (Mot), rapid forward movement (RFM), viability (PMI), hyposmotic swelling test (HOST) of frozen-thawed cat epididymal spermatozoa (n = 18 pools) in a commercial extender without (G0) and with three concentrations of vitamin E (G0.3, G0.6 and G0.9 m).

Group	Mot (%)	RFM	PMI (%)	HOST (%)
Fresh	75.0 ± 6.1^a^	3.55 ± 0.51^a^	74.5 ± 1.8^a^	76.7 ± 1.9^a^
G0	15.8 ± 1.8^b,c^	2.50 ± 0.14^b,c^	38.9 ± 2.5^b^	49.3 ± 1.9^b^
G0.3	20.3 ± 2.5^b^	2.77 ± 0.17^b^	40.3 ± 2.8^b^	52.4 ± 3.9^b^
G0.6	14.7 ± 1.9^b,c^	2.44 ± 0.16^b,c^	40.0 ± 2.5^b^	45.3 ± 3.0^b^
G0.9	12.7 ± 1.8^c^	2.11 ± 0.17^c^	38.0 ± 3.0^b^	48.5 ± 2.8^b^

a,b,c Different superscripts within columns are significantly different at P < 0.05.

The score for progressive motile spermatozoa (RFM) of fresh samples ranged from 3.0 to 4.0 (3.55 ± 0.51) and was significantly different from those of the frozen samples. The addition of α-tocopherol did not improve progressive movement, but spermatozoa from group 0.3 presented higher scores after thawing than did group 0.9 ( Tab. 1 ).

### Morphology

The percentage of morphologically normal spermatozoa in fresh samples was 64% ( Tab. 2 ). After thawing, this percentage for all groups (group 0, group 0.3, group 0.6 and group 0.9) ranged from 41.4 to 47.5%. There was a remarkable increase in the proportion of spermatozoa with tail abnormalities after thawing in all groups, but significant difference was only found with regards to fresh samples ( Tab. 2 ). In contrast, compared to fresh samples the percentage of proximal and distal droplets has decreased after thawing in all groups ( Tab. 2 ).

**Table 2:** Percentage of morphological abnormal spermatozoa obtained from cat epididymis (n = 18 pools) before (fresh) and after freezing-thawing process (G0, G0.3, G0.6, and G0.9).

Abnormalities	Fresh (%)	G0 (%)	G0.3 (%)	G0.6 (%)	G0.9 (%)
Minor head abnormalities	3.8^a^	4.05^a^	3.79^a^	3.61^a^	3.3^a^
Abnormalities midpiece implantation	0.83^a^	0.83^a^	0.94^a^	0.33^a^	0.5^a^
Minor tail abnormalities	9.2^b^	24.3^a^	25.05^a^	29.4^a^	27.03^a^
Distal droplets	8.6^a^	0.39^b^	0.16^b^	2.28^b^	0.27^b^
Major head abnormalities	2.1^a^	2.4^a^	1.5^a^	1.83^a^	1.6^a^
Midpiece defects	0.78^a^	1.27^a^	0.94^a^	0.4^b^	0.3^b^
Major tail abnormalities	8.5^b^	20.9^a^	19.7^a^	20.7^a^	24^a^
Proximal droplets	2.19^a^	0.39^b^	0.4^b^	0.05^b^	0.1^b^
Teratogenic defects	0^a^	0.16^a^	0.05^a^	0^a^	0.2^a^
Coiled tail with droplet	0	0	0	0	0
Normal	64^a^	45.33^a^	47.5^a^	41.4^a^	42.7^a^

a,b Different superscripts within rows are significantly different at P < 0.05.

### PMI and HOST

Data for PMI and HOST are presented in Table 1 . After thawing all groups exhibited a significant reduction in PMI compared to the values found in fresh samples (74.5 ± 1.8%). The mean value of PMI was not different (P > 0.05) among the post-thaw groups.

The percentage of spermatozoa that reacted to HOST was significantly lower in post-thawed samples compared to the fresh ones. There were no significant differences among the groups supplemented with α-tocopherol and the one without the antioxidant (G0, P > 0.05).

### Lipid peroxidation reaction

Data about TBARS are presented in Table 3 . The results showed no difference between control (group 0) and group 0.3, and among group 0.3, group 0.6 and group 0.9, with slightly lower values in group 0.6 and group 0.9.

**Table 3:** Mean (± standard deviation) values of lipid peroxidation using TBARS test in frozen-thawed cat epididymal spermatozoa (n=18 pools) after addition of three different concentrations of vitamin E. FR=Free radical production, GS=Maximum production and TBARS production = Antioxidant power.

Groups	FR	GS	TBARS production
G0	0.037 ± 0.007^a^	0.383 ± 0.092^a^	0.345 ± 0.070ª
G0.3	0.037 ± 0.006^ab^	0.341 ± 0.027^ab^	0.275 ± 0.109^ab^
G0.6	0.032 ± 0.006^b^	0.282 ± 0.078^b^	0.239 ± 0.074^b^
G0.9	0.032 ± 0.004^b^	0.305 ± 0.108^b^	0.251 ± 0.104^b^

a,b Different superscripts within columns are significantly different at P < 0.05.

## Discussion

Many studies with mammals (rabbits, horses, bovine, dogs, boar and ram) investigate the role that vitamin E plays in semen quality and report conflicting results ( Baumber *et al*., 2002 ; Castellini *et al*., 2003 ; Breininger *et al*., 2005 ; Hatamoto *et al*., 2006 ; Satorre *et al*., 2012 ; Towhidi and Parks, 2012 ; Silva *et al*., 2013 ; Belala *et al*., 2016 ). In domestic cats, supplementation of trolox, a water soluble vitamin E analogue, to the egg yolk Tris-Equex medium improved progressive motility, membrane integrity and DNA protection in epididymal semen of domestic cats after thawing, but no protective effect was observed on the acrosome membrane integrity ( Thuwanut *et al*., 2008 ). Michael *et al*. (2007) added 0.3 mM α-tocopherol to the Tris medium and reported no satisfactory results. The isoform of vitamin E, α-tocopherol, was used in this study because it has a good plasma membrane activity, protecting against lipid peroxidation ( Donnelly *et al*., 1999 ). However, Lopes *et al*. (2010) reported improved motility rates and acrosome preservation after thawing when adding 0.6 mM of the α-tocopherol to the Tris medium for dog semen. In the present study, the addition of α-tocopherol did not have the expected protective effect, since different sperm parameters (sperm motility, viability, and membrane functionality) did not differ between the supplemented and control group.

Significant differences between parameters of fresh and frozen-thawed semen were not surprising, since it is generally known that during cryopreservation different cryoinjuries affect spermatozoa. Instead, an effect of the presence of vitamin E was expected. However, in all vitamin E groups motility and forward progressive motility were lower than the values reported by Thuwanut *et al*. (2008) , when the Tris medium was supplemented with 5mM of trolox, a water soluble vitamin E. These differences can be attributed to the different media used (Botucrio^®^ *vs* . Tris) and the vitamin E isoforms (trolox *vs*. α-tocopherol).

Post-thaw sperm viability (evaluated by plasma membrane integrity test) was similar in all supplemented groups and it was not statistically different from the frozen group without vitamin E. Likewise, the post-thaw values given by the HOST did not differ between groups. It is important to underline that the only study reporting the HOST in feline species, although in ejaculated semen ( Comercio *et al*., 2013 ), used a 50 mOsmol/kg of fructose solution for 30-40 min of incubation. In the present study, we used epididymal semen which was incubated in 150 mOsmol of fructose and sodium citrate solution for 60 min. Although there is no other data available in the literature for comparison, the result was similar to that of the viability test (using eosin yellow dye) and so the HOST using these concentrations of fructose and sodium citrate was considered valid to assess plasma membrane integrity of cat spermatozoa and might be an alternative for routine evaluation of quality of frozen-thawed sperm of this species.

The percentage of different epididymal sperm abnormalities was also similar in groups frozen with and without vitamin E. Minor and major tail abnormalities were greater in frozen than in fresh samples, but the percentage of spermatozoa with proximal and distal droplets decreased after thawing. Axnér *et al*. (2004) reported lower percentage of spermatozoa with distal droplets and higher percentage of tail abnormalities in fresh samples diluted in Phosphate Buffered Saline (PBS) prior to fixation for morphological evaluation. According to those authors mixing epididymal spermatozoa with PBS or seminal fluid might promote changes in osmolarity and pH that induce dissolution of the droplet from the sperm midpiece and promote coiling of the tail. Taking this into account, it is possible that the extender used in the present study had an osmotic and/or pH influence on epididymal spermatozoa after thawing, resulting in lower percentage of droplets and higher tail abnormalities. As there is no information available regarding pH and osmolality of cat epididymal fluid, further research is needed to determine these parameters and how it might or not influence sperm morphology.

Another consideration about the morphological abnormalities found after thawing concerns the temperature and the time used for this step. The protocol for thawing at 70°C per 8 seconds was based on the study of Cocchia *et al*., (2009) . Better results were achieved by Thuwanut *et al*. (2008) with thawing at 70°C per 6 sec for cat epididymal semen frozen with Tris and trolox ( Thuwanut *et al*., 2008 ).

The TBARS evaluation shows the oxidative stress to which the sperm is exposed before and during the freezing process and as the vitamin E plays a role as a potent lipid peroxidation inhibitor (through the induction of oxidative stress with acid reactions). We had expected that the addition of this antioxidant would reduce lipid peroxidation reaction (TBARS production). High rates of lipid peroxidation after thawing are related to low motility rates and reduction in integrity of membrane, acrosome and DNA of sperm cells ( Thuwanut *et al*., 2010 ). In the present study, the addition of α-tocopherol to the BC extender did not present antioxidant effect; α-tocopherol concentrations of 0.6 and 0.9 mM, which resulted in higher rates of peroxidation through TBARS test, showed the worst values for RFM and Mot. It is possible that as BC was not able to protect cat epididymal cells from cryostress, adding α-tocopherol to this extender resulted in no antioxidant power.

Thus, we concluded that the addition of α-tocopherol to the commercial extender did not enhance post-thaw semen parameters. A possible explanation might be that the Botucrio® does not protect domestic cat’s epididymal cells from cryostress, and because of this, the addition of α-tocopherol could not provide its full antioxidant potential. Therefore, further research on the cryopreservation procedures with the use of other antioxidants for the epididymal semen of felids is recommended in order to develop the best protocol to preserve the genetic material of this species.
